# A Systematic Review of HIV Pre-exposure Prophylaxis (PrEP) Implementation in U.S. Emergency Departments: Patient Screening, Prescribing, and Linkage to Care

**DOI:** 10.1007/s10900-023-01320-7

**Published:** 2023-12-21

**Authors:** Kristopher J. Jackson, Pooja Chitle, Sandra I. McCoy, Douglas A.E. White

**Affiliations:** 1 Center for AIDS Prevention Studies, University of California, San Francisco, 550 16th Street – 3rd Floor, San Francisco, CA 94158 USA; 2 School of Public Health, University of California, Berkeley, CA USA; 3 Alameda Health System, Highland Hospital, Oakland, CA USA

**Keywords:** HIV, Differentiated service delivery, Pre-exposure prophylaxis, PrEP, Emergency department, Emergency medicine

## Abstract

In the pursuit of ending the HIV epidemic, U.S. emergency departments (EDs) have emerged as a valuable setting to increase HIV testing and linkage to care. There is limited data available, however, describing the incorporation of HIV prevention initiatives in U.S. EDs. Over the last decade, HIV pre-exposure prophylaxis (PrEP) has significantly changed the HIV prevention landscape globally and very little is known about the provision of PrEP in U.S. EDs. To address this gap in the literature, we conducted a systematic review of peer-reviewed quantitative studies and conference abstracts spanning July 2012 - October 2022. Of 433 citations, 11 articles and 13 abstracts meet our inclusion criteria, representing 18 unique studies addressing PrEP screening, prescribing, and/or linkage to PrEP care.

Most studies describe screening processes to identify PrEP-eligible patients (*n* = 17); most studies leveraged a patient’s STI history as initial PrEP eligibility screening criteria. Fewer studies describe PrEP prescribing (n = 2) and/or linkage to PrEP care (*n* = 8).

Findings from this systematic review highlight the potential for U.S. EDs to increase PrEP uptake among individuals at risk for HIV infection. Despite a growing number of studies exploring processes for incorporating PrEP into the ED setting, such studies are small-scale and time limited. Models providing prescribing PrEP in the ED show higher initiation rates than post-discharge engagement models. Electronic health record (EHR)-based HIV screening is valuable, but post-ED linkage rates are low. Our findings emphasize the need to establish best practices for initiating and supporting prevention effective PrEP use in the ED setting.

## Introduction

Emergency departments (EDs) often provide medical care to underserved and vulnerable patients at risk for HIV infection, many of whom utilize the ED as their primary or only source of health care [1]. Consequently, EDs present a unique opportunity to accelerate the process of ending the HIV epidemic. To date, the involvement of U.S. EDs in HIV prevention has primarily been to increase the identification of people living with undiagnosed HIV infection and linking these individuals to HIV care and treatment through integrated screening programs [2–7]. However, in some ED settings, it may also be logical to identify patients at risk of acquiring HIV infection and provide them with PrEP services, including education, and linkage to outpatient care.

### PrEP Eligibility Criteria and Same-Day PrEP

**Fig. 1:**
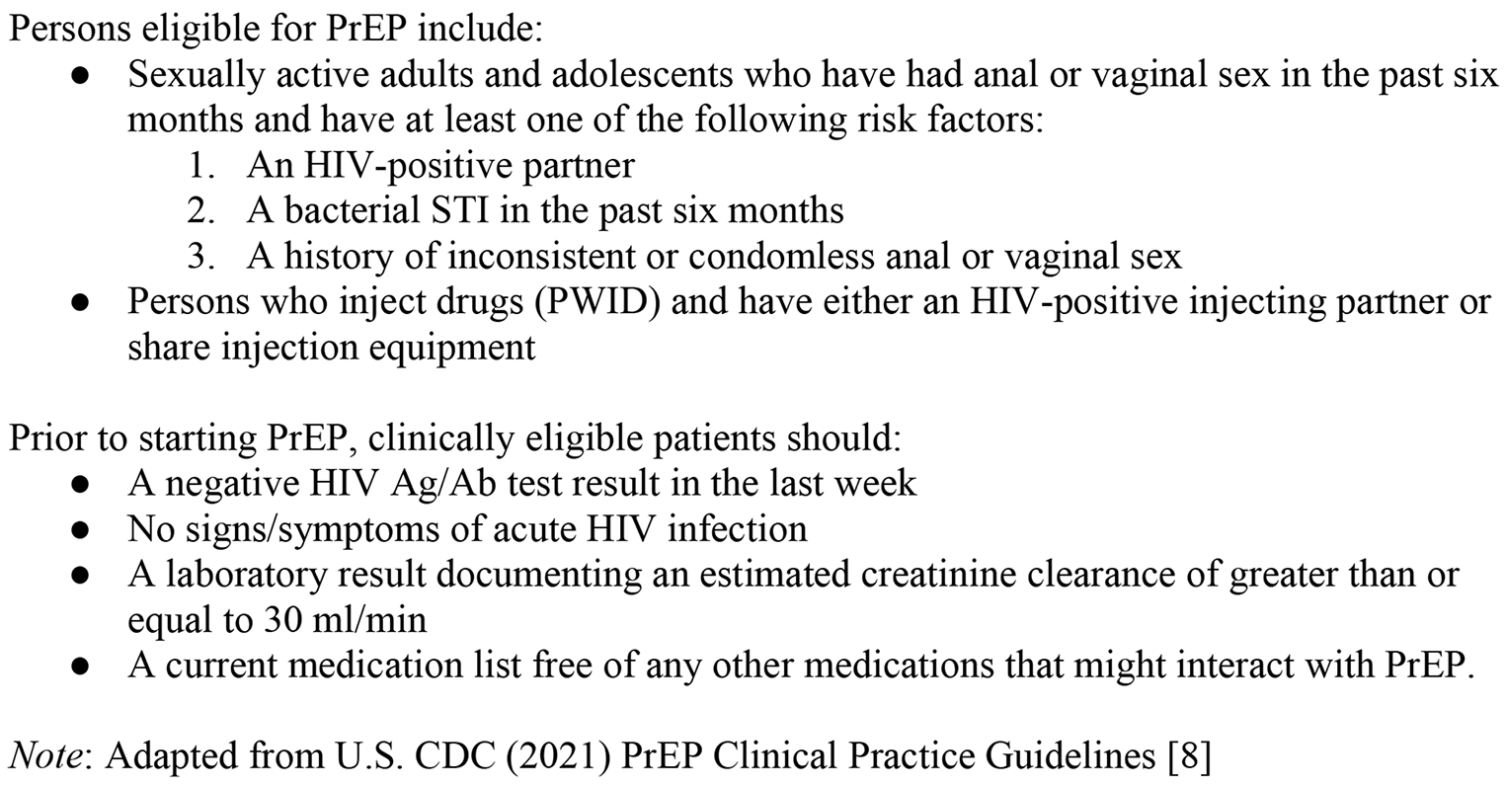
United States Centers for Disease Control HIV Pre-Exposure Prophylaxis (PrEP) Clinical Practice Guidelines (2020)

The 2021 U.S. Centers for Disease Control [8] clinical practice guidelines recommend PrEP for HIV-negative individuals at substantial risk of HIV acquisition, as outlined below in Fig. 1.

U.S. CDC (2021) clinical practice guidelines [8] also delineate an accelerated process for prescribing “same-day PrEP,” or PrEP prescribing in the context of a single clinical encounter and while awaiting some laboratory test results. Same-day PrEP eliminates barriers to PrEP access and has been shown to increase PrEP uptake and decrease time to PrEP initiation [9]. In this context, PrEP is prescribed to eligible individuals after a patient’s medical history is screened for: chronic hepatitis B virus (HBV) infection, pregnancy, indications for HIV post-exposure prophylaxis, and signs and symptoms consistent with acute HIV. When offering “same-day” PrEP, PrEP is prescribed to a patient *before* the results from traditional, “send-out” serum creatinine, HIV viral load testing, and/or HBV surface antigen testing results are available, typically within several days. Should a contraindication to PrEP use be identified based on these laboratory results, PrEP can be discontinued without discernible harm to the patient. While somewhat less common in clinical practice than the conventional PrEP initiation process spanning multiple encounters, same-day PrEP eliminates barriers to PrEP access and makes PrEP available at the time of an initial clinical consultation or at the time an indication for PrEP is identified in the clinical setting [9].

### PrEP and the Emergency Department

Individuals at risk for HIV acquisition routinely pass through U.S. EDs while seeking care for unrelated conditions [10–12]. Despite this, little is known about the feasibility of integrating PrEP into the ED setting. The integration of HIV prevention services in the ED, including PrEP, has until recently been an understudied approach to interrupting HIV transmission among people who could benefit from effective HIV prevention tools. Several proof-of-concept studies have demonstrated the feasibility of implementing various kinds of screening strategies in the ED to identify people who would benefit from PrEP, highlighting the promise of this approach to connect hard-to-reach individuals with HIV prevention services [11, 12]. However, the provision of PrEP in the ED setting poses several implementation challenges that could undermine organizational and ED provider willingness to adopt this strategy, including: a lack of provider knowledge and willingness to initiate PrEP, rigid clinical workflows that may inhibit adopting new processes/practices, a paucity of efficient, feasible, and reliable behavioral screening tools to identify subsets of higher-risk ED patients, and significant gaps in the understanding of the best clinical models for PrEP implementation (e.g., same day start vs. referral). Furthermore, the prospects for successful, long-term continuation of PrEP after ED-based initiation have yet to be described despite their centrality to the effectiveness and cost-effectiveness of this delivery channel to increase effective use of PrEP and ultimately interrupt HIV transmission. In addition to these barriers, there are important questions on the horizon about the optimal PrEP implementation model in the ED setting, including both the opportunities and barriers to offering long-acting, injectable PrEP (e.g., long-acting injectable cabotegravir) in U.S. EDs.

The purpose of this systematic review is to evaluate and synthesize the growing body of literature on ED-based PrEP implementation to identify area(s) where best practices may exist and where additional inquiry might be beneficial to better understand the role of ED-based PrEP as a differentiated service delivery model. We sought to focus our understanding of the scope of ED-PrEP service delivery on three key care cascade components: screening for PrEP eligibility, prescribing PrEP in the ED, and linkage to outpatient PrEP services.

## Methods

We conducted a systematic review of empirical studies of PrEP eligibility screening, prescribing, and/or linkage to PrEP care in U.S. EDs to summarize the body of evidence for ED-based PrEP service delivery and assess the feasibility of applying this evidence to clinical practice. We conducted a search of the PubMed, Cumulative Index to Nursing and Allied Health Literature (CINAHL), EMBASE, and Web of Science databases for English language, peer-reviewed articles published between July 2012 and October 2022. We selected this date range to capture all available publications since the July 2012 U.S. Food and Drug Administration (FDA) approval of the first combination medication for use as HIV chemoprophylaxis [13]. We limited each search to English-language quantitative research articles, conference abstracts, and gray literature that described the provision of PrEP services in the ED setting. We adopted broad search terms to ensure that any reference to the identification of PrEP-eligible patients, PrEP prescribing, and/or linkage to PrEP care in the ED setting were represented in our results. A list of the terms used for each of the four databases queried for this review are listed below:

- **PubMed**: ((HIV[Title/Abstract]) AND (“Pre-exposure prophylaxis“[Title/Abstract] AND “PrEP“[Title/Abstract])) AND (“Emergency“[Title/Abstract] AND “room“[Title/Abstract] OR “medicine“[Title/Abstract] OR “department“[Title/Abstract])
- **CINAHL**: HIV AND (“prep” AND “pre-exposure prophylaxis”) AND “emergency” AND (“department” OR “medicine” OR “room”).
- **Embase**: (‘hiv’/exp OR hiv) AND ‘pre-exposure prophylaxis’:ab,ti AND ‘prep’:ab,ti AND (‘emergency’:ab,ti AND ‘department’:ab,ti OR ‘room’:ab,ti OR ‘medicine’:ab,ti).
- **Web of Science**: ((AB=(HIV)) AND AB=(“PrEP” AND “Pre-exposure prophylaxis”)) AND AB=(“emergency” AND “medicine” OR “department” or “room”).

We excluded non-full text articles (except for peer-reviewed abstracts from published conference proceedings). We also excluded qualitative studies, editorials/commentaries, study protocols, systematic reviews, meta-analyses, as well as any non-peer-reviewed publications (e.g., theses or dissertations) from our results. We also excluded studies that did not describe any aspect of the PrEP care cascade, studies that used the ED for recruitment but did not involve any ED-provider-patient PrEP interactions. To ensure all relevant, contemporary discussions on the provision of PrEP were represented in this review, we also reviewed the reference lists of prior systematic reviews on this topic to identify potentially relevant works not returned by our selected search strategy [14, 15]. Our findings are presented in accordance with PRISMA-P guidelines; we registered the protocol for this review with the International Prospective Register of Systematic Reviews [16].

## Data Abstraction & Analysis

Articles and conference abstracts were screened independently by two reviewers (DW, KJ) using Covidence [17]. Any discrepancies in inclusion/exclusion coding were resolved by consensus. This process was repeated during full text review. Two reviewers (KJ, PC) independently completed the data abstraction process using a standardized extraction worksheet developed for this review (KJ, SM). For each included study or conference abstract, we abstracted the following data: study design, study location, aspect(s) of PrEP care addressed by the research (PrEP eligibility screening, PrEP prescribing and/or PrEP linkage).

For studies describing PrEP eligibility screeing, we additionally abstracted data describing the study population and the PrEP eligibility criteria adopted for each study. Notably, given the vastly different study designs and screening strategies in each study, it was not feasible to accurately calculate the proportion of each ED study population that was eligible for PrEP. In terms of PrEP linkage, we collected data pertaining to the study population, the process for linking individuals to outpatient PrEP services, the reported number and/or percentage of study patients linked to PrEP care, and whether PrEP was dispensed by a provider at the referral clinic. We operationalized ED PrEP prescribing as the issuance of a prescription for PrEP by an emergency medicine provider at the time of an ED visit, either evidenced by the number of PrEP prescriptions written by ED providers in the context of an ED visit and/or a description of the processes surrounding the issuance of a PrEP prescription. Due to the substantial heterogeneity in study designs, setting, and measures, we conducted a systematic narrative synthesis to summarize study characteristics and findings [18].

## Results

**Fig. 2:**
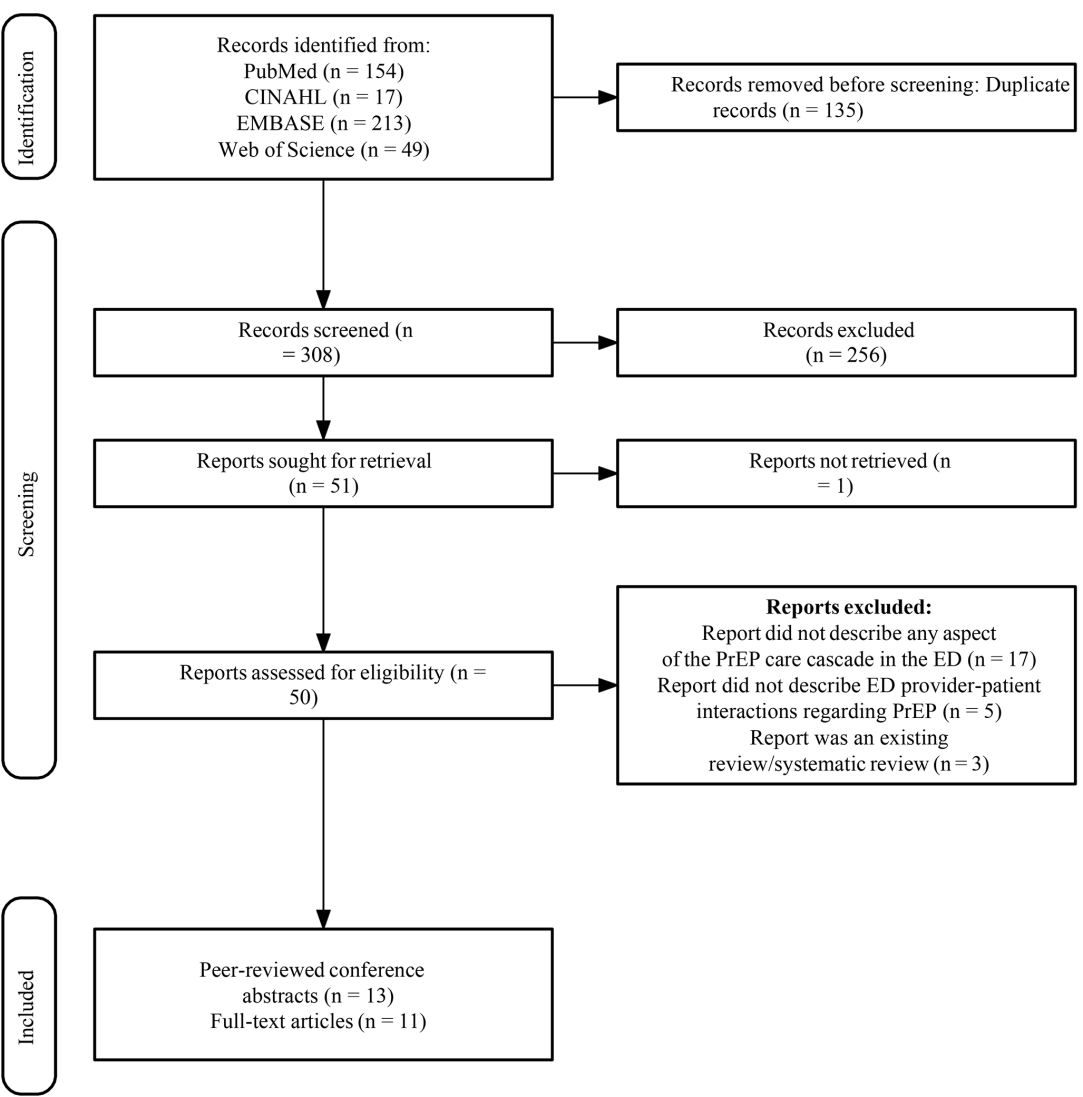
PRISMA flow diagram

Our search yielded 433 citations across the PubMed (*n* = 154), CINAHL (*n* = 17), Embase (n = 213), and Web of Science (n = 49) databases. After filtering duplicate entries (*n* = 135), we reviewed 308 abstracts for possible inclusion and 51 citations were identified for full text review (Fig. 2). Studies were excluded based on four criteria: (1). the study did not describe any aspect of the PrEP care cascade in the ED (*n* = 17), (2). the study used ED for recruitment, but no ED provider-patient PrEP interactions were described (*n* = 5), (3). the study was an existing systematic review (*n* = 3), and (4). the full text of article was unavailable (*n* = 1). Twenty-four works remained in our final sample, including 13 peer-reviewed conference abstracts and 11 peer-reviewed journal articles, reporting on 18 unique studies [19–42].

### Characteristics of Included Studies

As shown in Table 1, we identified 18 unique studies that quantitatively assessed PrEP eligibility screening, prescribing and/or linkage to care in the ED setting. All study designs were observational (Table 1), including: retrospective cohort studies (*n* = 6), cross-sectional studies (*n* = 5), prospective cohort studies (*n* = 6), and one multiple cohort study.

**Table 1:** Scholarly works describing three key components of the PrEP care cascade for U.S. ED patients: PrEP screening, prescribing, and linkage to continued PrEP care (July 2012- October 2022)

*Reference Number*	*Author(s) and Year*	*Study Type*	*Study Location*	*Entry Type (Article/Abstract/Both)*	*PrEP Screening*	*PrEP Prescribing*	*PrEP Linkage*	*Conference/Publication*
[19]	Carlisle et al. (2022)	Retrospective cohort study	Birmingham, AL	Article	■			*AIDS Patient Care and STDs*
[20]	Faryar et al. (2022)	Cross-sectional study	Cincinnati, OH	Article	■			*American Journal of Emergency Medicine*
[21–23]	Haukoos et al. (2022)	Cross-sectional study	Denver, CO Oakland, CA	Article and Conference Abstracts	■			*American Journal of Emergency Medicine* Society of Academic Emergency Medicine (2020); Two abstracts
[24]	Hazra et al. (2022)	Prospective cohort Study	Chicago, IL	Conference Abstract	■		■	Conference on Retroviruses and Opportunistic Infections (CROI) 2022
[25, 26]	Mahal et al. (2022)	Retrospective cohort study	New York City, NY	Article and Conference Abstract	■		■	*American Journal of Emergency Medicine* Annals of Emergency Medicine Research Forum (2020)
[27, 28]	McLaughlin et al. (2022)	Retrospective cohort study	New York City, NY	Article and Conference Abstract	■		■	*International Journal of STD & AIDS* STI & HIV World Congress (2019)
[29]	Haukoos et al. (2021)	Cross-sectional study	Denver, CO Oakland, CA	Article	■			*AIDS Patient Care and STDs*
[30]	Musoke et al. (2021)	Prospective cohort study	Northeast Ohio	Conference Abstract	■		■	ID Week 2021
[31]	Ridgway et al. (2021)	Multiple cohort study	Chicago, IL	Article	■			*AIDS Patient Care and STDs*
[32, 33]	Zhao et al. (2021)	Prospective cohort study	Baltimore, MD	Article and Conference Abstract	■		■	*Sexually Transmitted Diseases* STI & HIV World Congress (2019)
[34]	Ketels et al. (2020)	Prospective cohort study	Denver, CO	Conference Abstract	■		■	STD Prevention Conference (2020)
[35, 36]	Kulie et al. (2020)	Cross-sectional study	Washington DC	Article and Conference Abstract	■			*AIDS Patient Care and STDs* Society of Academic Emergency Medicine (2020)
[37]	Ridgway et al. (2018)	Prospective cohort study	Washington DC	Article	■			*AIDS Education and Prevention*
[38]	Ridgway et al. (2018)	Prospective cohort study	Washington DC	Article	■		■	*AIDS Patient Care and STDs*
[39]	Powell et al. (2019)	Cross-sectional study	Washington DC	Conference Abstract	■		■	Society of Academic Emergency Medicine (2019)
[40]	Magnuson et al. (2018)	Retrospective cohort study	United States	Conference Abstract		■		International AIDS Society Annual Meeting (2018)
[41]	Pitts et al. (2018)	Retrospective cohort study	New York City	Conference Abstract	■	■		ID Week (2018)
[42]	Reddy et al. (2020)	Retrospective cohort study	Davis, CA	Conference Abstract	■			ID Week (2020)

Table 1 lists the peer-reviewed journal in which each study was published and/or the conference at which each conference abstract was presented. Notably, each of the studies included in this review has been published in peer reviewed journals belonging to one of two distinct disciplines or research areas: emergency medicine or HIV/infectious disease. Notably, no study described all three cascade outcomes, and most 17/18 (94%) described screening; followed by linkage 8/18 (44%) and ED PrEP initiation 2/18 (11%).

### PrEP Eligibility Screening

The works in this review differ considerably with respect to study design, the data collected, and the definition of HIV risk – in fact, many of the studies operationalized risk in a manner different than the U.S. CDC clinical practice guidelines in place at the time the studies were conducted. The CDC (2017) clinical practice guidelines for PrEP eligibility were less streamlined prior to being updated in 2021 (see Fig. 1). Under the CDC (2017) clinical practice guidelines, HIV risk and PrEP eligibility among MSM was based self-reporting of an HIV + sexual partner, “recent” bacterial STI, a “high” number of sex partners, a history of inconsistent/no condom use, and/or reported commercial sex work [43]. Among heterosexual men and women, the same criteria applied – with the caveat that a given individual was “in a high prevalence area or network” (p. 13). Among persons who inject drugs (PWID), patients were considered PrEP eligible under U.S. CDC (2017) clinical practice guidelines if they endorsed having an HIV + injecting partner and/or reported sharing injection equipment. Sixteen of the 18 studies selected for inclusion describe some sort of PrEP eligibility screening process using many of the elements of the 2017 Clinical Practice Guidelines for PrEP (See Table 2). Nearly all included studies (*n* = 15) leveraged a patient’s STI history, current STI symptoms, or a diagnostic test result documenting an STI as a criterion for assessing possible PrEP eligibility, in accordance with CDC criteria for PrEP screening (Table 2). Other eligibility criteria, such as reports of inconsistent condom use (*n* = 6) and/or sex with a serodiscordant partner (*n* = 6) were often measured via dialogue between patients and providers in the context of eliciting a sexual history from a patient presenting with STI symptoms. Similarly, individuals seeking care in the ED with toxicology results or other EHR data suggesting ongoing/recent illicit drug use were consequently also asked social history questions about needle sharing, another common eligibility criteria (*n* = 6). Less common screening criteria were sensitive, specific questions about a patient’s sexual activity, such as a patient’s number of sexual partners over a specified period of time (*n* = 2), engaging in sex with PWID (*n* = 2), and/or belonging to high-prevalence HIV networks (*n* = 1).

**Table 2:** PrEP eligibility screening measures adopted in U.S. emergency departments (July 2012 - October 2022)

		PrEP Screening Criteria									
*Article*	*Study Population*	*Bacterial STI*	*Personal history of illicit drug Use♦*	*Inconsistent condom use*	*Sex with serodiscordant partner*	*PWID and shares needles/equipment*	*Sex with PWID who shares equipment*	*Commercial sex work*	*MSM sexual contact*	*Number of sexual partners*	*High HIV prevalence area/network*
Carlisle et al. (2022)	EHR data of HIV-negative patients treated at the University of Alabama (Birmingham) ED 2019 through 2020	■	■								
Faryar et al. (2022)	HIV-negative patients with positive risk screening in an urban academic ED in Cincinnati (OH)/Northern Kentucky 3/2019 through 2/2020	■		■	■	■	■		■		
Haukoos et al. (2022)	Denver Medical Health Center (Denver, CO) and Highland Hospital (Oakland, CA) patients who screened negative for HIV and were identified as high-risk for HIV.	■		■	■	■		■			
Hazra et al. (2022)	University of Chicago ED patients redirected from the ED for evaluation in a sexual wellness clinic (SWC) 2/2019-9/2019	■									
Mahal et al. (2022)	Jacobi Medical Center (Bronx, NY) ED patients who were HIV-negative, presented with STI complaints 1/2019-11/2019	■	■	■	■	■		■			
McLaughlin et al. (2022)	EHR data of HIV-negative patients treated at Bellevue Hospital ED (New York, NY) diagnosed with gonorrhea, chlamydia and/or syphilis 1/2014 through 7/2017	■									
Musoke et al. (2021)	Northeast Ohio VA Healthcare System patients diagnosed with a bacterial STI 3/ 2021 through 5/ 2021.	■									
Ridgway et al. (2021)*	University of Chicago ED patients with a negative HIV test in the ED 5/2018 through 8/2018	■							■		
Zhao et al. (2021)	Johns Hopkins ED (Baltimore, MD) patients who were HIV-negative with recent STI 12/2018 through 3/2019	■									
Ketels et al. (2020)	University of Colorado ED (Denver, CO) patients who were HIV-negative with bacterial STI diagnosis 3/2019 through 12/2019	■									
Kulie et al. (2020)	Washington DC ED (1 tertiary academic hospital, one public community hospital) patients who were HIV-negative, sexually active within the past 6 months, and presented with STI, substance use, or intentional injury-related complaints 1/2019 through 7/2019	■	■	■	■	■		■			
Ridgway et al. (2018)*	University of Chicago ED patients who were HIV-negative and completed an HIV-risk assessment with results indicating HIV risk 8/2015 through 11/2017	■							■		
Ridgway et al. (2018)	University of Chicago ED patients who were HIV-negative and underwent HIV prevention counseling 7/2015 through 8/2017	■		■	■	■			■	■	
Powell et al. (2019)	George Washington University Hospital ED (Washington D.C.) and affiliated urgent care (UC) patients who were HIV-negative and presented with genitourinary complaints	■		■	■	■	■	■		■	■
Pitts et al. (2018)	EHR data of HIV-negative patients treated. NYU Langone (New York City, NY) 1/2017 through July 2017.	■									
Reddy et al. (2020)	EHR data of HIV-negative UC Davis patients diagnosed with a bacterial STI 1/2017 -12/2017	■									
**Total (%)**		16 (94%)	3 (20%)	6 (38%)	6 (38%)	6 (38%)	2 (13%)	4 (27%)	4 (27%)	2 (13%)	1 (6%)
♦ = Toxicology screening positive and/or patient endorses history of drug use											

* = Patients screened for PrEP eligibility using an institutional HIV risk score developed to include the following elements: age, gender, gender of sexual partner, chief complaint, and positive test for sexually transmitted infection in the prior 6 months

*Abbreviation Key*:

MSM = Men who have sex with men

PWID = People who inject drugs

STI = Sexually transmitted infection

Four feasibility studies [19, 27, 28, 41, 42] used retrospective electronic health record (EHR) data to establish PrEP eligibility based upon past positive STI test results. Carlisle et al. (2022) used two slightly different approaches to PrEP eligibility screening in contrast to the other two projects that also used retrospective EHR searches [19]. Carlisle et al. (2022) considered patients PrEP eligible if the EHR for a given patient contained past positive result for trichomonas infection in addition to prior chlamydia, gonorrhea, and/or syphilis infections, whereas other studies only considered the latter. Further, patients were classified as PrEP eligible if toxicology data suggested patients were using heroin, amphetamines, cocaine, and/or other opiates. In prior studies, patients were only classified as PrEP eligible in this regard if they reported needle sharing or sexual practices with drug-using partners. In addition to retrospective review of EHR data, the EHR has also been used to identify patients at risk for HIV in real time. Musoke et al. (2021) and Ridgway et al. (2021) describe a process of programming the EHR to automatically flag patient records containing one or more HIV risk factors [30, 31]. These flags remind providers in the ED and/or other practice settings that a given patient might benefit from a conversation about PrEP during a future clinical encounter.

### ED PrEP Prescribing

Two conference abstracts described PrEP prescribing in the ED setting [40, 41]. Pitts et al. (2018) describes a retrospective review of patient medical records data (*N* = 1142) spanning ~ 43 months (January 2014 – July 2017) at an urban New York City hospital to determine the extent to which HIV-negative patients diagnosed with chlamydia, gonorrhea or syphilis were subsequently prescribed PrEP [41]. EHR data reviewed for this study were not strictly drawn from patients evaluated in the ED, with ED patients comprising 31.3% (*n* = 358) of patients diagnosed with an STI. Among patients in the sample evaluated in the ED for an STI (n = 358), 1.7% of the sample (*n* = 6) was prescribed PrEP by an ED provider.

Magnuson et al. (2018) conducted a study of U.S. prescription claims data from at least 80% of U.S. pharmacies, examining patterns of PrEP prescribing to adolescents ages 12–24 [40]. The authors found that among U.S. adolescents ages 12–17 prescribed PrEP between January and December 2017 (*n* = 2590), 83.5% (*n* = 2162) were female and 59% (*n* = 1528) were Medicaid recipients. Further, 21% of PrEP prescriptions in this population (*n* = 544) were issued by emergency medicine providers. Among individuals ages 18–24 during this same time frame, U.S. PrEP recipients (*n* = 24,740) were 75.3% (*n* = 18,635) were male and 22% (*n* = 5443) were Medicaid recipients. In this population, emergency medicine providers accounted for 12% of PrEP prescriptions (*n* = 2969) whereas 39% PrEP prescriptions originated from family medicine providers (*n* = 9649).

Because these two works are published conference abstracts based on retrospective data, no information is available about the processes used for prescribing PrEP, patient counseling, or whether any PrEP prescriptions furnished ED providers were issued as “same-day PrEP” or in the context of post-discharge follow-up (e.g. subsequent patient contact for follow-up, disclosure of diagnostic or laboratory test results, etc.).

### Linkage to Continued PrEP Care

As shown below in Table 3, eight of the included studies reported some measurement of linkage to PrEP care. Nearly all eight studies were executed in large, urban, academic medical center emergency departments. Hazra et al. (2022) and Mahal et al. (2022) report the highest rates of PrEP linkage and share a very important implementation element: same-day appointments with PrEP providers [24–26]. Both studies scheduled appointments with PrEP providers during patients’ ED visits and, when possible, conducted the visit with the PrEP provider in the context of or immediately following each patient’s ED encounter.

**Table 3:** PrEP linkage derived from treatment by U.S. emergency medicine providers (July 2012- October 2022)

*Author(s)*	*Study Population*	*Sample Size*	*Process for establishing linkage to PrEP care (if described)*	*% of patients linked to PrEP care (if described)*	*% of patients continuing PrEP (if prescribed)*
Hazra et al. (2022)	University of Chicago ED patients redirected from the ED for evaluation in a sexual wellness clinic (SWC) 2/2019-9/2019	*N* = 560	● Patients identified in the ED or after being notified of a positive STI result from ED provider ● Patients given prescription for PrEP by SWC provider ● Arranged for future primary care follow-up after SWC visit	● Same-day PrEP initiated in 16.1% (*n* = 90) patients	● 20% (*n* = 18) continued to take PrEP at 3 months; 11.1% (n = 10) continued to take PrEP at 6 months
Mahal et al. (2022)	Jacobi Medical Center (Bronx, NY) ED patients who were HIV-negative and presented with STI complaints 1/2019-11/2019	*N* = 1174; Linkage to PrEP measured for sub-sample (*n* = 22)	● Patient scheduled for follow-up with PrEP provider during ED visit ● PrEP provider visit scheduled (same-day if evaluated in ED during business hours)	● 100% of (*n* = 22) patients had follow-up appointment scheduled during ED visit ● 59% (*n* = 13) of patients referred attended PrEP provider visit and 11 of these patients were subsequently prescribed PrEP.	
McLaughlin et al. (2022)	Bellevue Hospital ED (New York, NY) patients who are HIV-negative and diagnosed with bacterial STI 1/2014 through 7/2017	*N* = 383	● None (observational study)	● 1.6% of patients (*n* = 6) prescribed PrEP during follow-up visit within 90 days of STI diagnosis ● Among patients *not* prescribed PrEP (n = 377), 79% did not have follow-up within 90 days of ED visit	
Musoke et al. (2021)	Northeast Ohio VA Healthcare System patients diagnosed with a bacterial STI 3/ 2021 through 5/ 2021.	*N* = 42	● Patients’ medical record flagged with STI testing/PrEP recommendation to trigger referral by VA health system providers	● 14% (6/42) received a PrEP referral; No patients started PrEP by the end of the study period.	
Zhao et al. (2021)	Johns Hopkins ED (Baltimore, MD) patients who were HIV-negative with recent STI 12/2018 through 3/2019	*n* = 162	● Patient referred to PrEP provider if amenable to referral and subsequent patient contact was made for scheduling	● Of the (*n* = 15) patients successfully scheduled for a PrEP follow-up visit; 3% (*n* = 2) patients completed an appointment	
Ketels et al. (2020)	University of Colorado ED (Denver, CO) patients who were HIV-negative with bacterial STI diagnosis 3/2019-12/2019	*n* = 157	● Patient referred to PrEP provider if amenable to referral ● Patient contacted after ED visit for scheduling and subsequent evaluation by PrEP provider	● 10.8% (*n* = 17) of patients referred were scheduled for PrEP provider visit ● Of the 17 patients scheduled, 7 patients attended the visit and 3.1% of the patients referred (*n* = 5) started PrEP	● No patients remained on PrEP at six months
Ridgway et al. (2018)	University of Chicago ED patients who were HIV-negative and completed an HIV-risk assessment with results indicating HIV risk	*n* = 51	● Patients who completed screening were referred for appointment with PrEP provider and, if indicated, prescribed PrEP.	● Of the 68.6% of patients (*n* = 35) interested in PrEP, 17.6% (*n* = 9) scheduled an appointment with a PrEP provider ● 7.8% of patients (*n* = 4) from the original sample initiated PrEP after an appointment with a PrEP provider	
Powell et al. (2019)	George Washington University Hospital ED (Washington D.C.) and affiliated urgent care (UC) patients who were HIV-negative and presented with genitourinary complaints	*N* = 151 (PrEP eligible *n* = 53)	● PrEP eligibility screening and subsequent referral by ED/UC provider.	● 46% of patients were amenable to referral for consideration of PrEP by PrEP provider	

While the outcome measures in these two studies differ slightly, Hazra et al. (2022) report a same-day PrEP initiation rate of 16.1% (*n* = 90) of 560 patients evaluated for PrEP in an ED-affiliated sexual wellness clinic during the study period spanning February 2020 through September 2021 [24]. However, this study only reported the total number of patients evaluated and the number of patients who initiated same-day PrEP. Thus, one cannot infer what proportion of patients treated in this sexual wellness clinic were considered PrEP eligible. Over half of the patients offered same-day PrEP in this setting were cisgender women and more than two-thirds of the patients treated in the sexual wellness clinic were Medicaid patients (*n* = 379).

In a retrospective observational study of potentially PrEP eligible patients evaluated in the ED meeting criteria for an ED-based HIV prevention and navigation program (*N* = 1174), Mahal et al. (2022) reported that 1.9% of PrEP-eligible patients (*n* = 22) interested in PrEP were scheduled for visits with a PrEP provider after their ED visit during an eleven-month study period spanning January 2019 through November 2019. Of the patients (*n* = 22) scheduled with a PrEP provider, 13 patients followed up and 11 patients ultimately started PrEP [25, 26].

The linkage to care outcomes described in the preceding two studies differ from the studies conducted by Ketels et al. (2020), Ridgway et al. (2018), and Zhao et al. (2021) wherein ED providers generated PrEP referrals and generally relied on subsequent patient contact to schedule an appointment with a PrEP provider in the future [32–34, 38]. Between March through December 2019, Ketels et al. (2020) describes a sample of patients evaluated in an urban Colorado ED diagnosed with gonorrhea, chlamydia, trichomonas, and/or bacterial vaginosis (*N* = 289) who were offered a follow-up call after their ED visit from a PrEP coordinator to discuss HIV prevention when contacted with STI test results [34]. More than three-fourths (*n* = 224) of this predominantly female sample (*n* = 215) was under 35 years of age. Of the patients contacted by ED staff, 54.3% of patients (*n* = 157) agreed to be contacted by a PrEP coordinator. After speaking with a PrEP coordinator, more than 89% (n = 140) of patients declined a PrEP clinic referral. Of the relatively few (*n* = 17) patients for whom a PrEP appointment was scheduled, only seven attended their clinic visit and five patients ultimately started PrEP – representing only 1.7% of the original sample. No patients in the Ketels et al. (2020) study remained on PrEP at six months [34]. Interestingly, only Ketels et al. (2020) and Hazra et al. (2022) reported patients’ rate(s) of continued PrEP use following PrEP initiation following evaluation by an emergency medicine provider [24, 34]. Also shown in Table 3, Hazra et al. (2022) found that 20% of study patients (*n* = 18) continued to take PrEP at three months and 11.1% of study patients (*n* = 10) continued to take PrEP at six months post-initiation [24].

Using the EHR data of patients treated in a Washington DC ED, Ridgway et al. (2018) piloted the use of an electronic risk scoring system to guide HIV prevention counseling and PrEP initiation efforts, either during an ED visit or as part of post-encounter follow-up [37]. During the five-and-a-half-month pilot study period, 180 patients triggered an alert for HIV prevention services. Of these, the authors found that patients approached *during* their ED visit were more likely to complete HIV prevention counseling and risk assessment as compared to those contacted via phone after their visit (96.2%, 25/26 vs. 74.3%, 26/35, *p* = 0.02). Among patients who completed HIV risk assessment and counseling (*n* = 51), 68.6% (*n* = 35) of patients expressed interest in PrEP and nine patients were scheduled for an appointment with a PrEP provider. Of the nine patients who scheduled a PrEP appointment, three patients, or 1.6% of the original sample, initiated PrEP.

From December 2018 through April 2019, Zhao et al. (2021) describe a total of 314 ED patients evaluated in an academic medical center in Baltimore who were deemed potentially PrEP eligible based upon EHR data [32, 33]. Ultimately, 119 patients were approached about HIV prevention and possible PrEP use. Of these, 33% (*n* = 39) of patients expressed an interest in PrEP and were referred to peer navigators and less than half of these patients (*n* = 16) were scheduled for appointments with a PrEP provider. Of the 16 patients who were scheduled for an appointment with a PrEP provider, a total of four patients – approximately 3.4% of the total number of ED patients approached – ultimately started PrEP.

## Discussion

**Fig. 3:**
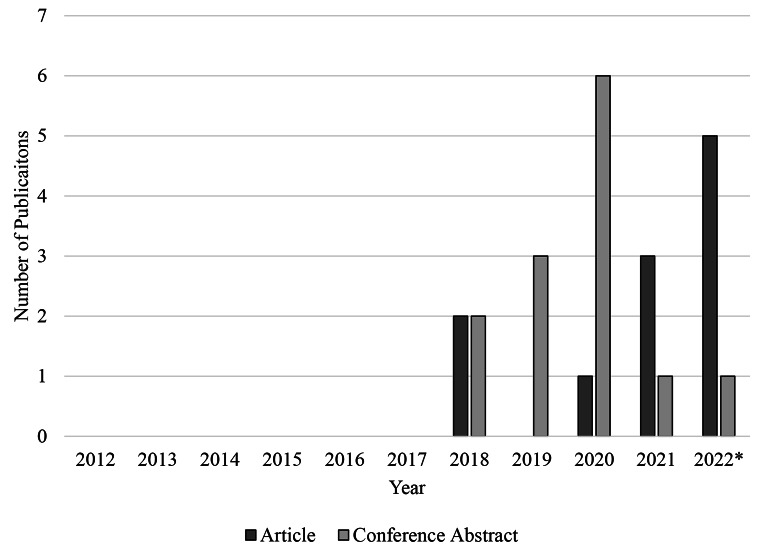
Peer reviewed articles and conference abstracts describing PrEP screening, PrEP prescribing, and/or linkage to PrEP care in U.S. emergency departments (July 2012 – October 2022)

Individuals at risk for HIV-infection routinely seek care in U.S. EDs. This systematic review suggests there is growing interest in incorporating PrEP as an HIV prevention strategy in U.S. EDs among both U.S. emergency medicine providers and members of the HIV/AIDS prevention research community, though past demonstration projects have been small and limited in duration. Figure 3 displays the publishing trends of peer reviewed studies and conference abstracts meeting criteria for inclusion in this review, spanning from the FDA approval of the first medication approved for use as PrEP in 2012 through October 2022, suggesting increasing interest regarding the feasibility of incorporating PrEP as an HIV prevention strategy in the ED setting. The decline in the number of publications describing PrEP eligibility, prescribing, and/or linkage to PrEP care observed in 2020 could be consistent with the onset of the COVID-19 pandemic.

The findings from this review suggest that the development of a patient-centered, PrEP implementation model in the ED setting may be feasible. There is not yet, however, an established “best practice” for: (1). identifying patients who might benefit from PrEP in the ED setting, (2). prescribing PrEP in the ED setting, and/or (3). linking patients to outpatient PrEP care.

In terms of patient screening for PrEP eligibility, leveraging EHR data to identify patients with active or recurrent STI symptoms or positive STI test results is a simple, commonly adopted method for readily identifying patients who may benefit from PrEP in a busy clinical setting. Many of the PrEP eligibility criteria adopted by the studies represented in this review, however, were adapted from U.S. CDC (2017) clinical practice guidelines and require providers to obtain complex social histories and inquire about highly sensitive topics. Provider rapport affects the reporting of this information accurately in most conventional clinical settings and may be even more difficult in the ED where time is limited and most providers are evaluating patients in the context of what are typically one-time clinical encounters in loud, hectic spaces with limited privacy. Social desirability may motivate patients who engage in sex work, use illicit drugs, or those who identify as a sexual and/or gender minority to conceal aspects of their identity in clinical environments where they feel judged or stigmatized by new and/or unknown providers.

Though our review includes peer-reviewed conference abstracts which have been excluded in prior systematic reviews on the provision of PrEP in the ED setting [14, 15], there is still a paucity of studies describing PrEP prescribing in the ED setting, either in the form of post-discharge follow-up care or offering patients same-day PrEP. All studies that were included in our review were published in specialized journals from the fields of emergency medical or HIV/infectious disease, suggesting that findings from academic inquiry into the feasibility or ability to identify, prescribe, and/or link patients to PrEP care in U.S. EDs are being circulated predominantly in niche publications instead of the broader allied health, medicine, nursing, and public health research communities. Additionally, the brief decline in published, peer-reviewed ED-based PrEP studies previously may have been the result of shifting patient care priorities in the face of a pandemic that overburdened many healthcare delivery systems globally. The demand for COVID-19 associated research also may have significantly affected editorial and publishing priorities for many peer-reviewed journals during this time [44].

The limited data presented in this review (which, notably, did not include randomized control trials) suggests that PrEP prescribing is, in fact, occurring in U.S. EDs. Additional implementation-science oriented research is necessary to determine how to best ensure patients deemed as PrEP eligible in the ED are offered PrEP, provide low barrier access to the medication, and streamline referral pathways to long term outpatient comprehensive HIV prevention services.

The studies with the highest rates of linkage to PrEP care in this review [24–26] had one important element in common: same-day access to PrEP. Patients with same-day access to a PrEP provider (whether in the ED or an adjacent sexual wellness clinic) were more likely start PrEP. Studies such as Ketels et al. (2020), Ridgway et al. (2018), and Zhao et al. (2021) required ED providers to generate PrEP referrals and relied on subsequent patient contact to schedule an appointment with a PrEP provider at an undefined point in the future [32–34, 38]. Consistent with the findings of multiple intervention trials of patients with chronic conditions discharged from the ED or the hospital, patients referred for PrEP are more likely to follow-up if they have an appointment made at the time of discharge or have assistance in making a follow-up appointment [45–47]. Thus, for medical centers or health systems with in-house, affiliated PrEP providers, scheduling patients for an appointment prior to discharging a patient from the ED may represent a simple but effective method for improving both access and PrEP linkage. For patients prescribed same-day PrEP in the ED, appointment scheduling prior to discharge ensures patients have a secure plan for ongoing access to PrEP. This is particularly important given that some U.S. ED providers may be reluctant to prescribe PrEP without therapeutic bridge to a more permanent PrEP solution beyond an initial supply of PrEP furnished by an ED provider.

The limited data presented in this review on continuation of PrEP once patients are linked to PrEP care suggests that rates of PrEP continuation in this patient population may be low, although data on this indicator are limited. Additional research is necessary to determine how to ensure patients have ongoing access to PrEP once initiated in the ED setting, Further, additional research is needed to determine if there are any differences among patients screened for PrEP eligibility, prescribed PrEP, or referred for PrEP care in the ED as compared to alternative settings.

There is an urgent need for further implementation science research to explore what referral models are best to ensure that people who could benefit from PrEP and who are interested have access to quality, ongoing care. The provision of PrEP via community-based organizations, telehealth/telePrEP programs, and retail pharmacy partnerships are examples of novel PrEP access strategies currently being piloted in communities across the U.S.

While not the focus of this review, some emergency medicine providers may be reluctant to address PrEP in the context of an ED visit, particularly to medically complex patients or patients perceived as likely to be lost to follow-up. A given emergency medicine provider’s comfort level around ascertaining patients’ HIV risk, their perceptions of PrEP, and/or their PrEP knowledge may limit their willingness to discuss and offer PrEP to patients. Thus, additional training and resources are necessary to ensure that emergency medicine providers recognize PrEP as a safe and highly effective HIV prevention strategy worthy of consideration when treating patients at risk for acquiring HIV.

Although initiating same-day PrEP may reduce barriers to access, increase patient uptake, and is a practice supported by CDC guidelines, prescribing PrEP in the ED faces several implementation challenges, including a lack of knowledge and familiarity with PrEP among ED providers, a lack of requisite laboratory data recommended to start PrEP, a lack of reliable, established referral networks for patient follow-up, and concerns about prescription coverage and out of pocket costs to patients. Prospective studies evaluating the feasibility of same-day ED PrEP are needed, including patients who are at risk for HIV acquisition not just from sexual exposure but also patients who are at risk as a result of needle sharing. Further, the long-term outcomes of ED PrEP initiation and adherence are needed.

## Conclusion

In the U.S., emergency medicine providers conduct approximately 140 million patient encounters annually and provide care to the medically underserved, individuals experiencing poverty, and individuals living with substance use disorders [48]. U.S. EDs are strategically positioned not only to identify people with HIV infection, but are also well positioned to provide HIV prevention services, including PrEP. Further research into the best practices for integrating ED PrEP eligibility screening, prescribing, and linkage to care – ideally in the form of prospective comparative trials - are needed. Such research may lead to valuable, evidence-based interventions that could increase PrEP uptake among ED populations at the greatest risk for acquiring HIV – many of whom might otherwise not have access to or seek out HIV prevention services.

## Data Availability

All data relevant to the study are included in the article or included as a supplementary appendix.
